# Dendritic Cell Migration Toward CCL21 Gradient Requires Functional Cx43

**DOI:** 10.3389/fphys.2018.00288

**Published:** 2018-03-27

**Authors:** Richard Ruez, Juan Dubrot, Alice Zoso, Marc Bacchetta, Filippo Molica, Stéphanie Hugues, Brenda R. Kwak, Marc Chanson

**Affiliations:** ^1^Department of Pediatrics, Cell Physiology, and Metabolism, Geneva University Hospitals, University of Geneva, Geneva, Switzerland; ^2^Department of Pathology and Immunology, University of Geneva, Geneva, Switzerland

**Keywords:** dendritic cells, connexins, migration, chemotaxis, mice

## Abstract

Dendritic cells (DCs) travel through lymphatic vessels to transport antigens and present them to T cells in lymph nodes. DCs move directionally toward lymphatics by virtue of their CCR7 and a CCL21 chemotactic gradient. We evaluated *in vivo* and in bone marrow-derived dendritic cells (BMDCs) whether the gap junction protein Cx43 contributes to CCL21/CCR7-dependent DC migration in wild-type (WT) mice, heterozygous (Cx43^+/−^) mice and mice expressing a truncated form of Cx43 lacking its regulatory C-terminus (Cx43^K258/−^). In a model of flank skin inflammation, we found that the recruitment of myeloid DCs (mDCs) to skin draining lymph nodes was reduced in Cx43^K258/−^ mice as compared to WT and Cx43^+/−^ mice. In addition, the migration of Cx43^K258/−^ BMDCs toward CCL21 was abolished in an *in vitro* chemotactic assay while it was only reduced in Cx43^+/−^ cells. Both mutant genotypes showed defects in the directionality of BMDC migration as compared to WT BMDCs. No difference was found between the three populations of BMDCs in terms of expression of surface markers (CD11c, CD86, CD80, CD40, MHC-II, and CCR7) after differentiation and TLR activation. Finally, examination of the CCR7-induced signaling pathways in BMDCs revealed normal receptor-induced mobilization of intracellular Ca^2+^. These results demonstrate that full expression of an intact Cx43 is critical to the directionality and rate of DC migration, which may be amenable to regulation of the immune response.

## Introduction

Connexin43 (Cx43) is expressed in immune cells and its importance in critical processes such as cell migration, phagocytosis, antigen (Ag) presentation, T-cell reactivity and B-cell responses is increasingly recognized (Glass et al., 2015). Connexins form hexameric structures, called connexons, at the plasma membranes. Two connexons from neighboring cells can dock to form a gap junction channel. Although connexons also open to the extracellular space, many early reports have to be reinterpreted with the discovery of pannexins, which also form pannexon channels at the membrane and fulfill similar functions than connexons. The term hemichannels is then awkwardly used for both structures (Sosinsky et al., 2011). In the recent years, non-channel functions have emerged for Cx43, many of which are linked to cytoskeletal dynamics (Matsuuchi and Naus, 2013).

Dendritic cells (DCs) are primary mediators in shaping adaptive immune responses by sensing peripheral danger signals and migrating to draining lymph nodes where they activate naïve T-cells into cytotoxic T lymphocytes and T helper cells. Migration of mature DCs from peripheral tissues to lymph nodes is driven by the regulated expression of CCR7, the receptor for CCL19 (Epstein-Barr virus-induced receptor ligand chemokine) and CCL21 (secondary lymphoid tissue chemokine) (Dieu et al., 1998; Sallusto and Lanzavecchia, 2000). Interestingly, Cx43 is expressed by myeloid DCs (mDCs) and up-regulated by pathogen-associated molecular patterns (PAMPs), cytokine cocktails or after tissue injury (Matsue et al., 2006; Corvalàn et al., 2007). Despite this evidence, little is known on how Cx43 influences immune responses. Several *in vitro* studies reported that inhibition of gap junction channels blocked DC activation, suppressed Ag transfer between DCs or Ag presentation to T-cells (Neijssen et al., 2005; Matsue et al., 2006; Mendoza-Naranjo et al., 2007; Elqueta et al., 2009; Yu et al., 2016). In contrast, Mazzini and collaborators found in mouse DCs harboring conditional deletion of Cx43 normal expression of DC markers and normal *ex vivo* T-cell responses to both MHC class I and II restricted Ags (Mazzini et al., 2014). Rather, the authors concluded that Cx43 favored locally the transfer of Ags sampled by macrophages to DCs in the intestinal mucosa.

Here, we evaluated intralymphatic DC migration toward draining lymph nodes in a model of skin inflammation in mice heterozygous for Cx43 (Cx43^+/−^) and in mice expressing a truncated form of Cx43 that lacks its regulatory C-terminus (Cx43^K258/−^). We report that Cx43 regulates the migration of activated DCs to lymph nodes. Additional *in vitro* investigations using bone marrow-derived dendritic cells (BMDCs) confirmed that an intact Cx43 C-terminus is required for CCR7-driven DC migration toward a CCL21 gradient. CCL21-dependent mDCs migration was impaired by connexin channel but not by pannexin channel inhibition. We conclude that Cx43 is a component of the mechanisms regulating directed migration of stimulated DCs to secondary lymphoid organs.

## Materials and methods

### Mice

Mice heterozygous (Cx43^+/−^ mice) for the Cx43 gene (*GJA1*) were previously described (Reaume et al., 1995). Both WT and Cx43^+/−^ mice (B6;129SGja1m1Kdr/J) were purchased from Jackson Laboratory (Bar Harbor, ME, USA). Mice in which the C-terminal region of Cx43 was truncated at K258 (Cx43^K258/−^ mice) were bred as previously reported (Maass et al., 2004). Of note, homozygous expression of the null or truncated alleles of Cx43 is lethal (Reaume et al., 1995; Maass et al., 2004). All experiments were performed in series using littermates of similar age and weight and of both genders. Cx43^+/+^ littermates (WT mice) were used as controls. Mice were kept under standard housing conditions with a fixed light/dark cycle. The Swiss cantonal veterinary authorities approved animal experimentation.

### *In vivo* DC migration assay

*In vivo* DC migration was induced by a contact hypersensitivity assay, as described previously (Dubrot et al., 2014). In brief, a mix of 1:1 acetone/dibutyl phthalate was applied on the skin to the right flank of the mice for 24 h. Skin-draining lymph nodes were carefully ground and digested at 37°C in RPMI 1640 containing 1 mg/ml Collagenase IV (Worthington Biochemical Corporation, Lakewood, NJ), 40 μg/ml DNase I (Roche, Basel, Switzerland) and 2% FCS for 40 min, gently mixing the samples every 20 min. The reaction was stopped by adding a 10% FCS solution containing 5 mM EDTA (FACS buffer). Samples were then filtered using a 70 μm cell strainer, centrifuged 5 min at 1,300 rpm and resuspended in FACS buffer for flow cytometry staining with for B220, TCRβ, CD11c and MHCII Abs (BioLegend, San Diego, CA). Total number of DCs was counted and percentage of CD11c^Int^MHCII^hi^ DCs was determined to calculate the number of newly migrating DCs in lymph nodes (see Figure S1A). The proportion of T and B cells in the draining lymph nodes as compared to the non-draining ones was not changed (Figure S1B).

### Lymphatic drainage

Drainage of interstitial fluids was quantified using Evans Blue injections in the footpad of WT and Cx43^+/−^ mice, as described previously (Meens et al., 2017). In brief, mice were anesthetized and injected with 5 μl 5% Evans Blue (dissolved in PBS) in the left footpad using a micro syringe. After 15 min, blood was collected by puncturing the left ventricle and centrifuged 15 min at 5,000 rpm (4°C). Formamide (Sigma-Aldrich, St. Louis, MO) was added to each serum sample (500 μL formamide/200 μL serum) and the mix was incubated overnight at 55°C. Thereafter, presence of Evans Blue in the serum was quantified by measuring the fluorescence using a SpectraMax Paradigm Multi-Mode Microplate reader (excitation: 620 nm; emission 670 nm; Molecular Devices, Sunnyvale, CA).

### BMDC differentiation and activation

Bone marrow cells were isolated from WT, Cx43^+/−^ and Cx43^K258/−^ mice by flushing femurs and tibiae of posterior members, and seeded in Petri dishes in complete DMEM, supplemented with L-arginin (0.116 g/l), L-asparagin (0.036 g/l), pyruvic acid (0.11 g/l), 2-β-mercaptoethanol (0.05 mM), Glutamine (2 mM), Hepes (10 mM), Penicilin/Streptomycin (30 U/ml/30 mg/ml), FCS (10%) and GM-CSF (10 ng/ml, Bachem, Bubendorf, Switzerland). This medium was renewed every 3 days during 10–12 days to allow BMDC differentiation. LPS (200 nM), PolyIC (2 μM), or CpG (1 μM) was added for the last day of culture to activate BMDCs. To determine differentiation and activation, cells were harvested and resuspended in FACS buffer for flow cytometry staining with CD11c, CD86, CD80, CD40, MHC-II, and/or CCR7 conjugated Abs with various fluorophores. All Abs were obtained from BioLegend. For cell surface markers analysis, single cell suspensions were pre-incubated with FcBlock (anti-CD16/32 FcγRII-RIII, BD Pharmingen, Allschwil, Switzerland) at 4°C for 10 min, before adding Abs or isotype controls. Data were acquired with a Facscalibur flow cytometer (BD Pharmingen) at the Flow Cytometry facility of our Medical Faculty and analyzed using FlowJo software (FlowJo LLC).

### RNA extraction, RT-PCR and qPCR

Cellular RNA was extracted using Nucleospin RNA II kits (Macheley-Nagel, Oensingen, Switzerland). Reverse transcription was performed using Quantitect Reverse Transcription kits (Qiagen, Hilden, Germany) for qPCR with a UNOII PCR thermocycler (Biometra GmbH, Göttingen, Germany). qPCR were performed using primers and probes from TaqMan® (Thermo Fisher Scientific) on MicroAmp Fast Optical 96-Well Reaction Plate with Barcode (Thermo Fisher Scientific), in a StepOnePlus™ Real-Time PCR System (Applied Biosystems,Foster City, CA). Gene expression was normalized to GAPDH expression in order to calculate 2^−ΔCT^.

### *In vitro* BMDC migration assay

For cell migration tracking experiments, cells resuspended in RPMI 1640 supplemented with 10 mM HEPES and 10% FCS, were mixed with 1.6 mg/ml collagen I (PureCol®, Advanced BioMatrix, San Diego, CA) at the final density of 3 × 10^6^ cells/ml and directly seeded in the migration channels of a μ-Slide chemotaxis^3D^ chamber (Ibidi, Munich, Germany), in accordance to the manufacturer's instructions. The chambers at the left and right side of the migration channels were filled with complete culture medium, with or without adding the connexin inhibitor 18-α-glycyrrhetinic acid (αGA, 50–100 μM, Cayman Chemical, Ann Harbor, MA) and/or the pannexin inhibitor probenecid (2 mM, Molecular Probes, Eugene, OR) for 1 h. CCL21 (Peprotech, London, UK) was next added (100–200 ng/ml final titration) only to the left chamber to generate a chemotactic gradient. A trypan blue exclusion test revealed no difference in BMDC mortality after 3 h exposure to vehicle (DMSO), 2 mM Probenecid or 100 μM αGA.

Time-lapse microscopy was performed using the ImageXpress System (Molecular Devices, Sunnyvale, CA, USA), equipped with a 4x phase contrast objective. The μ-Slide chemotaxis^3D^ chambers loaded with BMDCs were inserted into the 37°C heating stage of the ImageXpress System and images were acquired at a rate of one frame per 3 min for 2–4 h. The ImageJ software (National Institutes of Health, Bethesda, USA) plugin “Manual Tracking,” included in the FIJI bundle, was used to perform cell tracking. For each independent experiment, at least 50 cells were monitored excluding immobile cells. The “Chemotaxis and Migration Tool V2.0” © Ibidi GmbH allowed further analysis, by providing different graphs and statistical tests. Exported tables from the “manual tracking” plugin, in the ASCII format, were directly run into this software. The cell tracks were all extrapolated to (x, y) = 0 at time point 0 h. For quantitative evaluation of directed cell migration, several parameters of the trajectories were measured to provide indications on how fast and straight cells move and how much this movement is directed toward the chemotactic gradient:

- the center of mass (COM) represents the averaged coordinates of all cell endpoints, at a given moment, for a number n of analyzed cells. Its x and y values (respectively parallel and perpendicular to the chemotactic gradient) indicate the direction in which the group of cells primarily moved; this parameter evaluates chemotaxis.
- the velocity represents absolute cell speed, whatever the direction. Velocity was calculated by dividing the accumulated distance by the time length of the cell tracking, for each cell. Values, which are expressed in μm/min, represent the average velocity of n cells for each condition.
- the forward migration indices (FMI) parallel and perpendicular to the gradient represent the efficiency of forward migration of cells in relation to the axis parallel and perpendicular to the chemotactic gradient, respectively. Values represent the average FMI parallel and perpendicular of n cells for each condition; a strong chemotaxis effect leads to a high parallel FMI (positive or negative) and a low perpendicular FMI.
- the directness represents a measure of how straightforward are cell trajectories, whatever the direction. This parameter was calculated by dividing the Euclidian distance by the accumulated distance for each cell. Values represent the average directness of n cells for each condition. This parameter does not evaluate chemotaxis directly; however, it can be influenced by chemotaxis.
- the Rayleigh test evaluates the uniformity of a circular distribution of points around a common origin. The null hypothesis “uniformity” is rejected with *p* > 0.05.

### T-cell proliferation assay

The ability of Cx43-deficient BMDCs to induce Ags-dependent T cell proliferation was tested as follows. CD4^+^ T cells were purified from spleen and lymph nodes of OT-II TCR transgenic mice using CD4^+^ T cell depletion kit and the MACS system (Miltenyi Biotec, Bergisch Gladbach, Germany). LPS was added overnight to BMDCs for the last day of maturation in the presence of GM-CSF. Activated BMDCs were then incubated with the MHCII-restricted OVA323-339 peptide for 30 min at 37°C, washed three times and then added to the OTII cell culture. Naive CFSE-labeled OT-II cells (5 × 10^4^) were co-cultured with 5 × 10^4^ BMDC for 6 days. At days 1, 2, 3, and 6, proliferation of CD4^+^ T-cells was determined by CFSE dilution in a Facscalibur.

### Ca^2+^ measurement

To monitor changes in cytosolic Ca^2+^ concentration [Ca^2+^]_i_, BMDCs were incubated in the presence of 5 μM Oregon-Green 488 BAPTA-1 AM (Molecular Probes), a calcium indicator, for 1 h at 37°C, while attaching to glass coverslips. Cells were washed with Hank's balanced salt solution (HBSS) supplemented with 1.3 mM CaCl_2_, 1 mM MgCl_2_, and 10 mM HEPES (pH 7.4) to remove probe excess. The coverslips were then transferred to the stage of an inverted fluorescence microscope (TMD-300; Nikon AG, Zurich, Switzerland). A peristaltic pump was used to superfuse HBSS supplemented with CCL21 (200 ng/ml). Fluorescent Oregon-Green–loaded BMDCs were viewed with a 63 × /1.25 oil Iris Plan-Neofluar objective lens (Zeiss, Feldbach, Switzerland). Images were captured every 5 s with a high sensitivity CoolSnap HQ2 camera (Visitron systems GmbH, Puchheim, Germany) using the Metafluor software (Visitron systems), and fluorescence intensity was measured from regions of interest defined on every cell in the field. Because Oregon-Green 488 BAPTA-1 AM is a single-wavelength dye, its emission is a function of both cytosolic Ca^2+^ concentration and dye concentrations. Cytosolic Ca^2+^ changes were therefore expressed as the F1/F0 ratio, where fluorescence intensity values (F1) are divided by the initial fluorescence intensity (F0) measured during the recording.

### Statistical analysis

GraphPad Prism (La Jolla, CA) software (version 4.03) was used to compare experiments using unpaired *t*-tests, one-way ANOVA and the non-parametric Mann Whitney *U*-test, where appropriate. Values are expressed as mean ± SEM. ^*^p < 0.05 was considered significant; ^**^p < 0.01; ^***^p < 0.001.

## Results

### mDC recruitment to thoracic lymph nodes is reduced in mice with altered Cx43 expression

**Figure 1:**
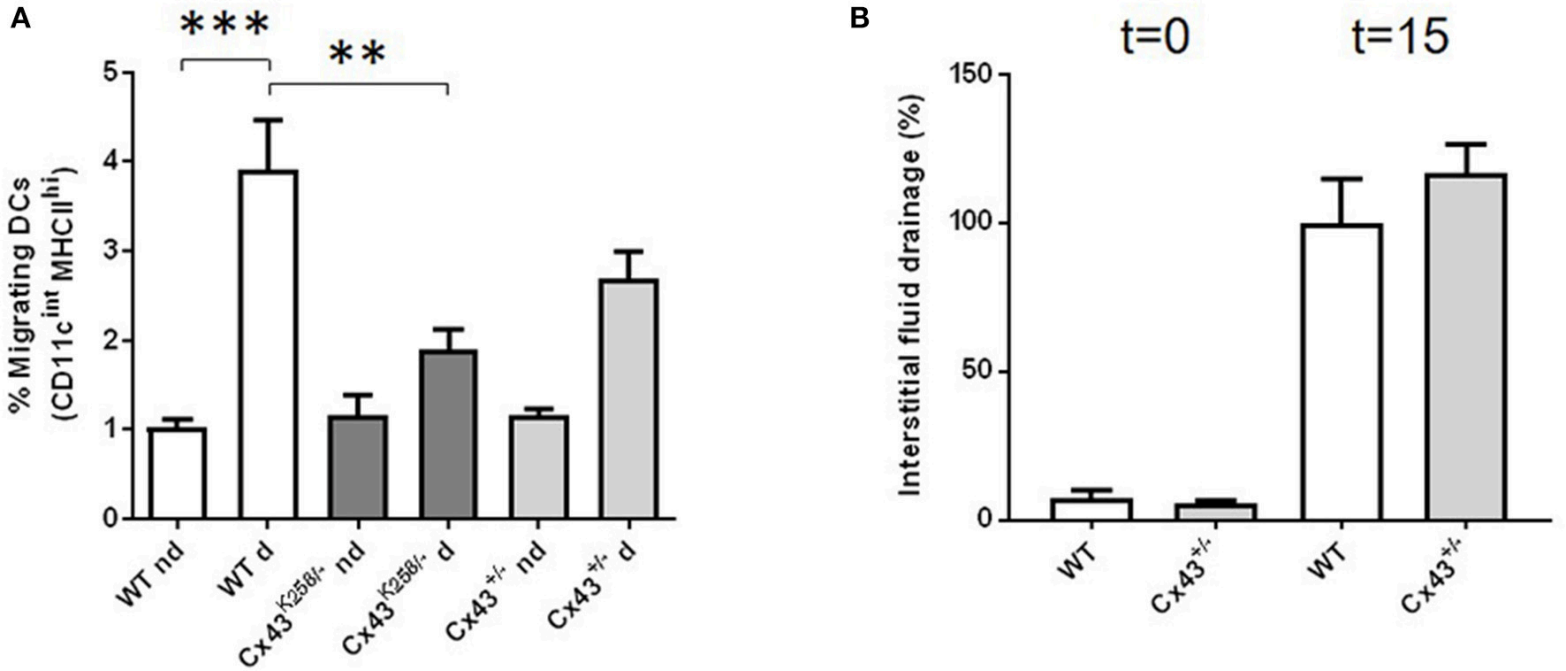
*In vivo* migration of mDCs to lymph nodes in mice with altered Cx43 expression. **(A)** 24 h after epicutaneous hapten sensitization of the right flank, the proportion of CD11c^Int^MHCII^hi^ in skin-draining (d) and in the contralateral flank control (nd) lymph nodes was measured in WT, Cx43^+/−^ and Cx43 Cx43^K258−^ mice. The proportion (%) of immigrating DCs to draining lymph nodes of Cx43^K258/−^ and Cx43^+/−^mice was decreased as compared to WT mice. *n* = 3–6 mice for nd lymph nodes and 5–7 for d lymph nodes. **(B)** No difference in the interstitial fluid drainage (%) was detected by Evans Blue recovery in the blood 15 min after its injection in the left footpad of Cx43^+/−^ and WT mice. *n* = 4–5 mice. ^*^p < 0.05 was considered significant; ^**^p < 0.01; ^***^p < 0.001.

The newly migrating DCs to lymph nodes can be discriminated from resident DCs by their CD11c and MHCII expression profile (CD11c^Int^MHCII^hi^). We have monitored DC expression markers in draining lymph nodes after skin inflammation in WT, Cx43^+/−^ and Cx43^K258/−^ mice. Twenty-four hours after application of acetone/dibutyl phthalate to the skin on the right flank, the percentage of CD11c^Int^MHCII^hi^ DCs detected in skin-draining draining lymph nodes (WT d) was increased as compared to control lymph nodes (WT nd) from the contralateral flank in WT mice (Figure 1A). In contrast, enhanced DC migration was not observed in Cx43^K258/−^ and Cx43^+/−^ mice, although the latter showed an intermediate behavior (Figure 1A). Importantly, the migration of DCs to draining lymph nodes of Cx43^K258/−^ mice was markedly decreased as compared to WT (Figure 1A).

The Cx43^+/−^ and Cx43^K258/−^ genotypes are, however, not specifically targeting DCs. Reduced DC migration in genetically modified mice might also result from decreased lymphatic flow induced by the ubiquitous deletion of one of the Cx43 alleles. We thus compared drainage of interstitial fluids following injection of Evans Blue in the left footpad of Cx43^+/−^ and WT mice. The dye, which progressively spreads throughout the lymphatic vessels to successive draining lymph nodes, is recovered in the blood. We collected sera 15 min after injection and the amount of dye was quantified. As illustrated in Figure 1B, Evans Blue transport to the systemic circulation was not different between WT and Cx43^+/−^ mice. These results suggest that the migration of DCs in mice expressing reduced amount of Cx43 or a truncated form of Cx43 is altered, and point to Cx43 as a potential novel partner in DC chemotaxis.

### BMDCs from mice with altered Cx43 expression show normal differentiation, activation, and ability to mediate T-cell response

**Figure 2:**
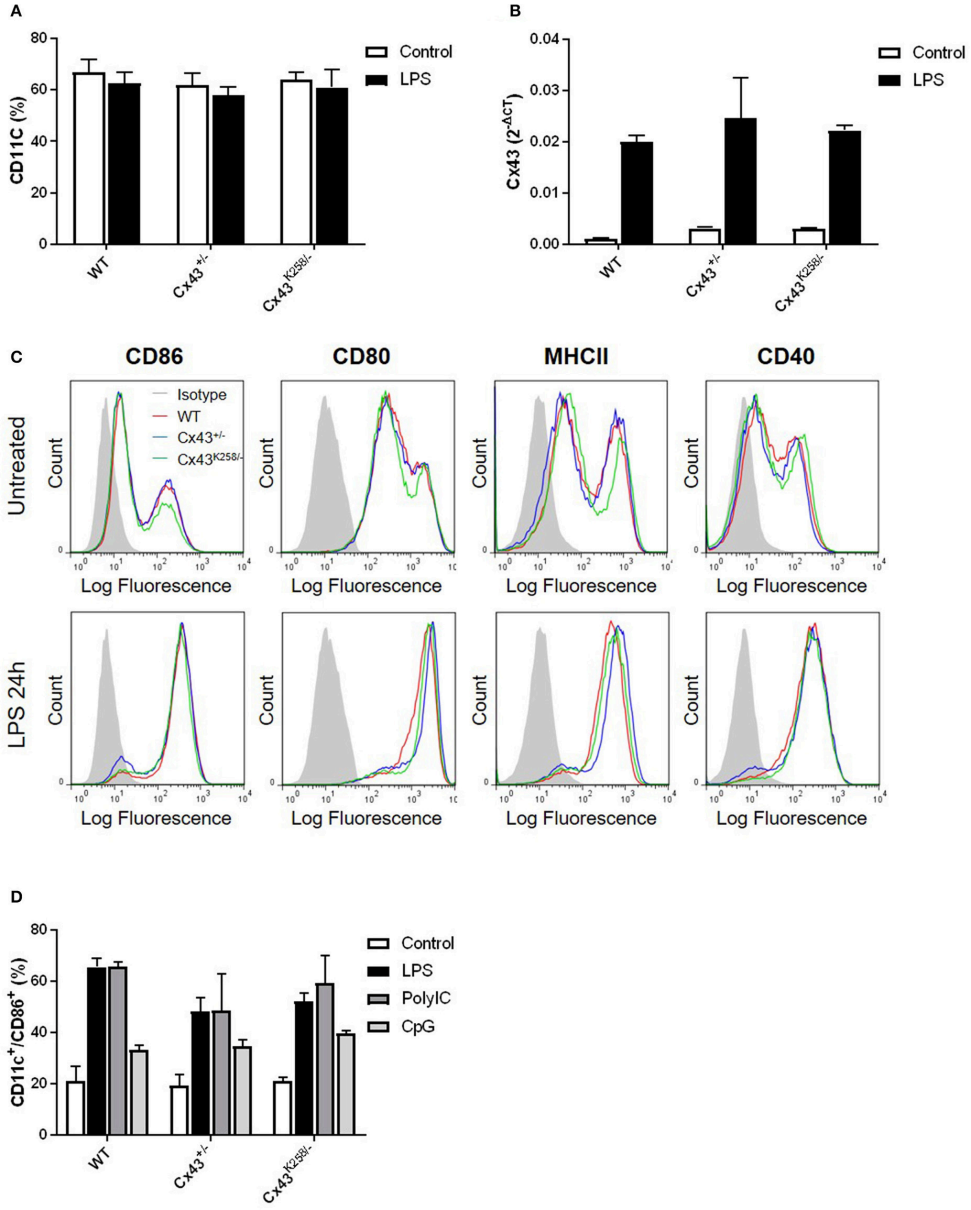
Differentiation and activation of BMDCs from mice with altered Cx43 expression. **(A)** The proportion (%) of CD11c-expressing bone marrow cells after *ex-vivo* treatment with GM-CSF for 12 days was similar between WT, Cx43^+/−^ and Cx43 Cx43^K258−^ mice with or without LPS activation. *n* = 7, 7 and 3 mice for WT, Cx43^+/−^ and Cx43^K258−^ BMDCs, respectively. **(B)** Cx43 mRNA is induced by a 24 h LPS treatment to similar levels in CD11c^+^ BMDCs from WT, Cx43^+/−^ and Cx43^K258−^ mice. Data are from a representative qPCR experiment performed on triplicate samples. **(C)** No difference in the expression of co-activation markers, including CD86, CD80, MHCII, and CD40 was observed between LPS-activated CD11c^+^ BMDCs from Cx43^+/−^ and Cx43 Cx43^K258−^ mice. Isotype controls performed on WT BMDCs are shown in gray; they are representative of isotype controls for each genotype. Independent FACS experiments comparing each genotype were performed for CD86 (*n* = 4), CD80 and CD40 (*n* = 1) or MHCII (*n* = 3). **(D)** TLR activation with either LPS, PolyIC or CpG induced similar proportion (%) of CD11c^+^/CD86^+^ cells in BMDCs obtained from WT, Cx43^+/−^ and Cx43^K258/−^ mice (*n* = 3).

To further investigate the mechanism by which Cx43 might affect DC migration, we differentiated bone marrow progenitor cells isolated from WT, Cx43^+/−^ and Cx43^K258/−^ mice to DCs (BMDCs) with GM-CSF. BMDCs were identified by FACS after staining for CD11c before and after activation with LPS for 24 h. As shown in Figure 2A, no difference in the percentage of CD11c-expressing cells was observed between WT, Cx43^+/−^ and Cx43^K258/−^ BMDCs. Cx43 expression was determined by qPCR in CD11c^+^ BMDCs activated or not with LPS (Figure 2B). LPS increased Cx43 mRNA expression not only in WT BMDCs, as previously reported (Matsue et al., 2006), but also in Cx43^+/−^ and Cx43^K258−^ BMDCs (Figure 2B). The activation of BMDCs by LPS was confirmed by increased expression of co-activation markers, including CD86, CD80, MHCII, and CD40 (Figure 2C). No difference in the expression of these markers was observed between WT, Cx43^+/−^ and Cx43^K258/−^ BMDCs (Figure 2C). We also compared the response of BMDCs to other PAMPs, such as PolyIC and CpG, and again observed no change in the proportion of CD11c^+^/CD86^+^ cells between WT, Cx43^+/−^ and Cx43^K258/−^ BMDCs (Figure 2D). These results indicate that reduced expression of Cx43 or expression of a truncated form of Cx43 did not affect the differentiation and the activation of BMDCs.

**Figure 3:**
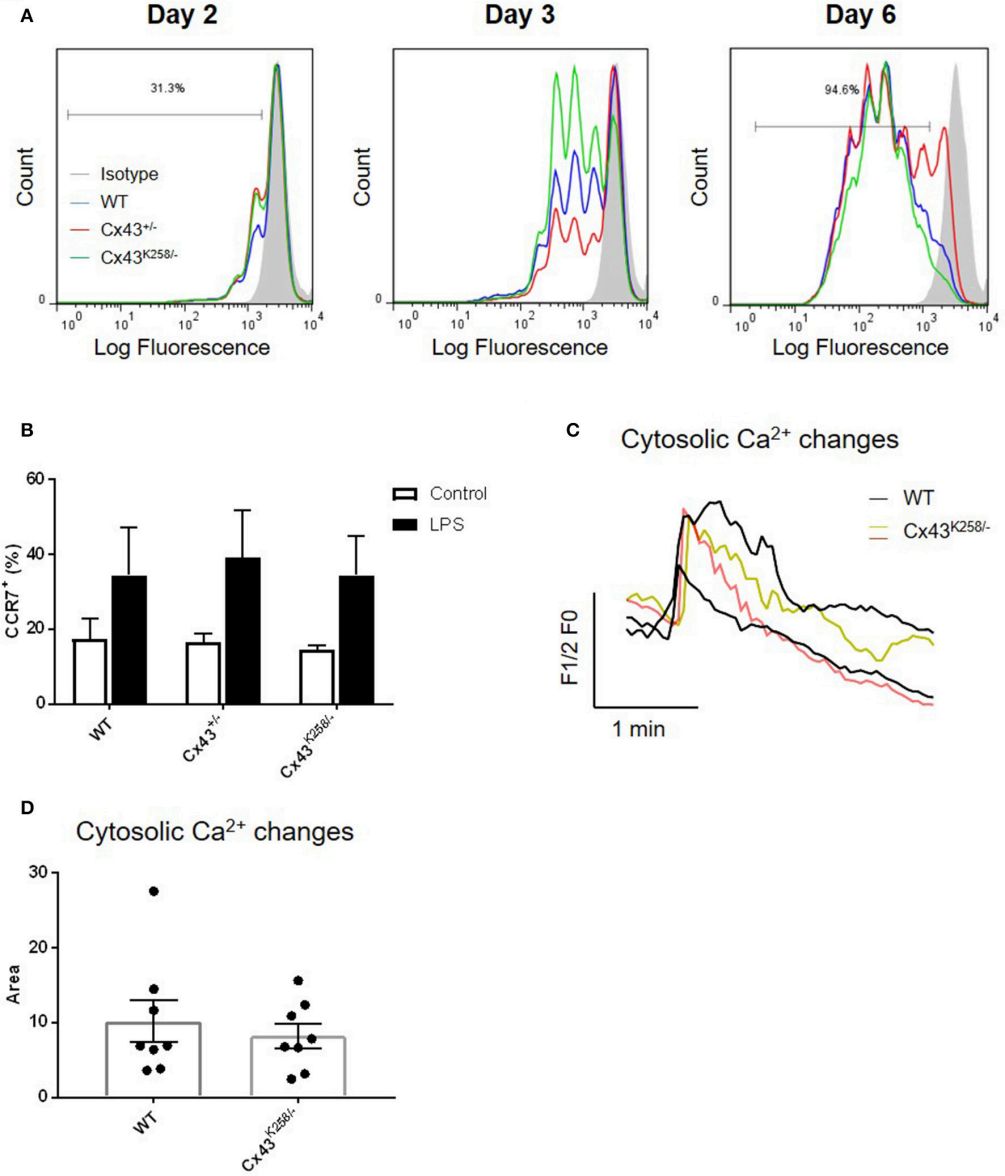
**(A)** T-cell proliferation assay showing similar response over time (day 2, 3, and 6) by FACS after co-culture of CFSE-labeled naïve T-cells with BMDCs from Cx43^+/−^ and Cx43 Cx43^K258/−^ mice pre-loaded with ovalbumine-related peptide. Data are representative of 2 independent experiments. **(B)** LPS activation of WT, Cx43^+/−^ and Cx43^K258/−^ BMDCs induced similar proportion (%) of CCR7^+^ BMDCs (*n* = 3). **(C)** Cytosolic Ca^2+^ changes evoked by CCL21 were monitored by time lapse imaging of Oregon-green loaded LPS-activated BMDCs. Rapid transient increase in [Ca^2+^]_i_ superposed by oscillations was detected in both WT (black lines) and Cx43^K258/−^ BMDCs (green and red lines). Traces from two WT and two Cx43^K258/−^ BMDCs are shown for comparison and clarity. **(D)** Area values measured under the [Ca^2+^]_i_ traces in multiple WT and Cx43^K258/−^ BMDCs that responded to CCL21.

The ability of presenting Ags to lymph node-resident T-cells is one of the most important functions of DCs. To determine whether BMDCs with altered Cx43 expression were able to induce a T-cell response *in vitro*, naïve CD4^+^/CD45.1^+^ cells labeled with the fluorescent dye CFSE were co-cultured with BMDCs that were pre-loaded with ovalbumine-related peptide, and T-cell proliferation induced by BMDCs of each genotype was monitored by FACS as a function of time. As shown in Figure 3A, Figure S2A, Cx43^+/−^ and Cx43^K258−^ BMDCs induced T-cell proliferation as efficiently as WT cells. These results demonstrate that alteration in Cx43 structure (Cx43^K258/−^ BMDCs) and expression (Cx43^+/−^ BMDCs) does not impair the ability of BMDCs to mediate T-cell proliferation *in vitro*.

CCR7 expression in activated BMDCs is critical for CCL21 chemotaxis, adhesion to and migration across the lymphatic endothelium. As shown in Figure 3B, Figure S2B, CCR7 was similarly expressed to the surface of WT, Cx43^+/−^ and Cx43^K258/−^ BMDCs after activation with LPS. To verify that engagement of CCR7 led to an intracellular signaling response in BMDCs, we monitored, by live imaging, changes in [Ca^2+^]_i_ in response to CCL21. CCL21 induced rapid mobilization of [Ca^2+^]_i_ followed by a progressive decline to baseline while superimposed oscillations could be observed in some cells in both WT and Cx43^K258/−^ BMDCs. Representative Ca^2+^ changes are illustrated in Figure 3C. To evaluate for differences in the [Ca^2+^]_i_ responses between WT and Cx43^K258/−^ BMDCs, we have measured the surface under the traces in multiple experiments; no difference was observed between BMDC of both genotypes in responses to CCL21 (Figure 3D). These results demonstrate that alteration in Cx43 structure and expression does not impair the ability of BMDCs to respond to CCR7 activation.

### BMDCs from mice with altered Cx43 expression have defective directed migration

**Figure 4:**
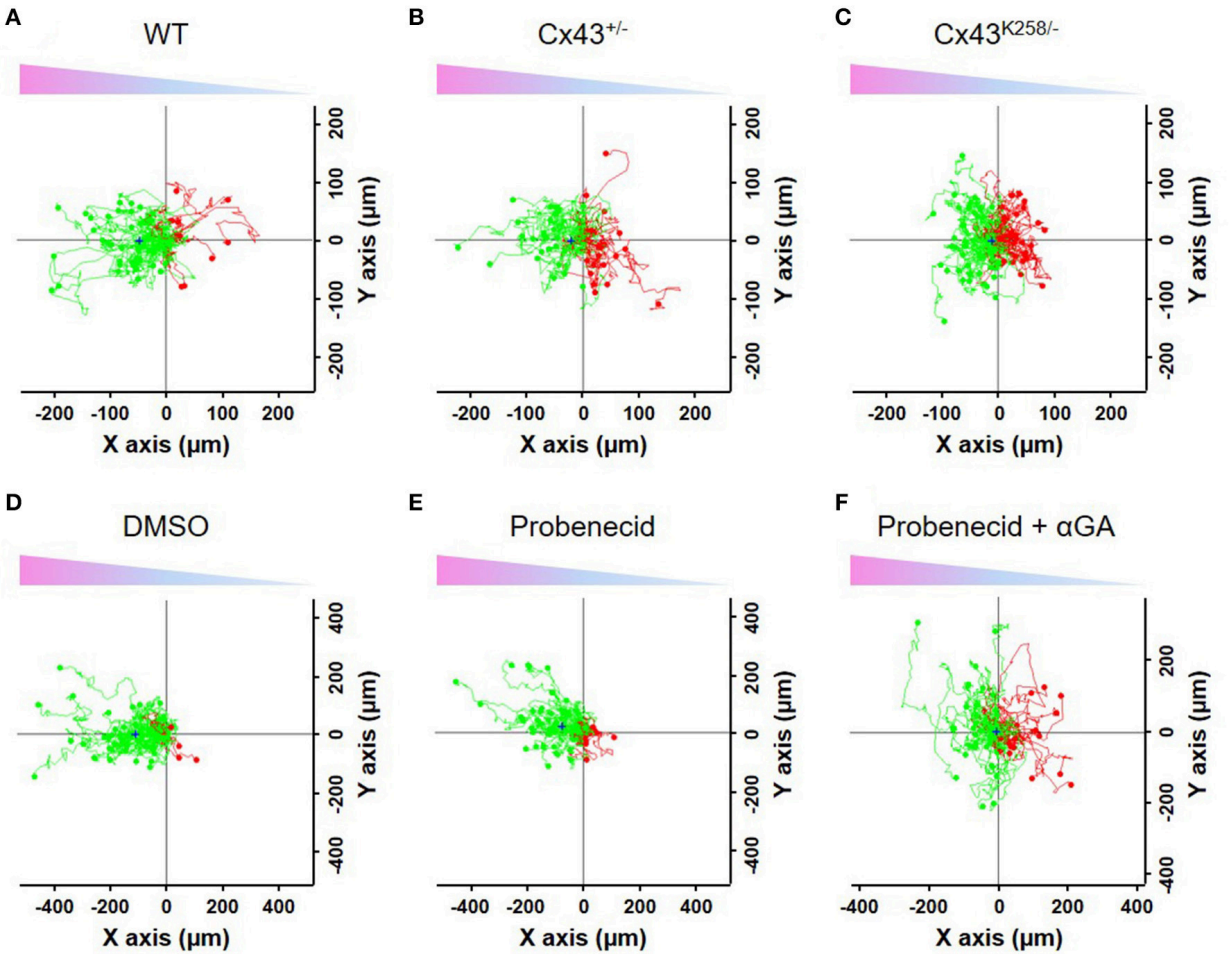
*In vitro* migration of LPS-activated BMDCs toward a CCL21 gradient. Migratory behavior of WT **(A)**, Cx43^+/−^ **(B)** and Cx43^K258/−^ **(C)** LPS-activated BMDCs monitored by time-lapse imaging for 3.5 h while subjected to a left to right (X axis) decreasing gradient of CCL21 (indicated by the arrows on top of the graphs). The trajectories of BMDCs that moved toward the CCL21 gradient are in green while the ones that moved against the gradient are in red. “+” symbol on graphs indicates the COM of the cell population at the end of the experiment. Alteration of Cx43 expression and structure perturbed the BMDCs' trajectories as compared to WT BMDCs. Effects of DMSO **(D)**, Probenecid **(E)** and αGA **(F)** on the migratory behavior of WT LPS-activated BMDCs. DMSO, which was used as vehicle, nor Probenecid (Prob), a pannexin1 channel inhibitor did prevent the BMDC migration toward the CCL21 gradient. In contrast, addition of the connexin channel inhibitor αGA (Prob+ αGA) blocked the directional movement of BMDCs. Probenicid: 2mM; αGA: 100 μM.

**Figure 5:**
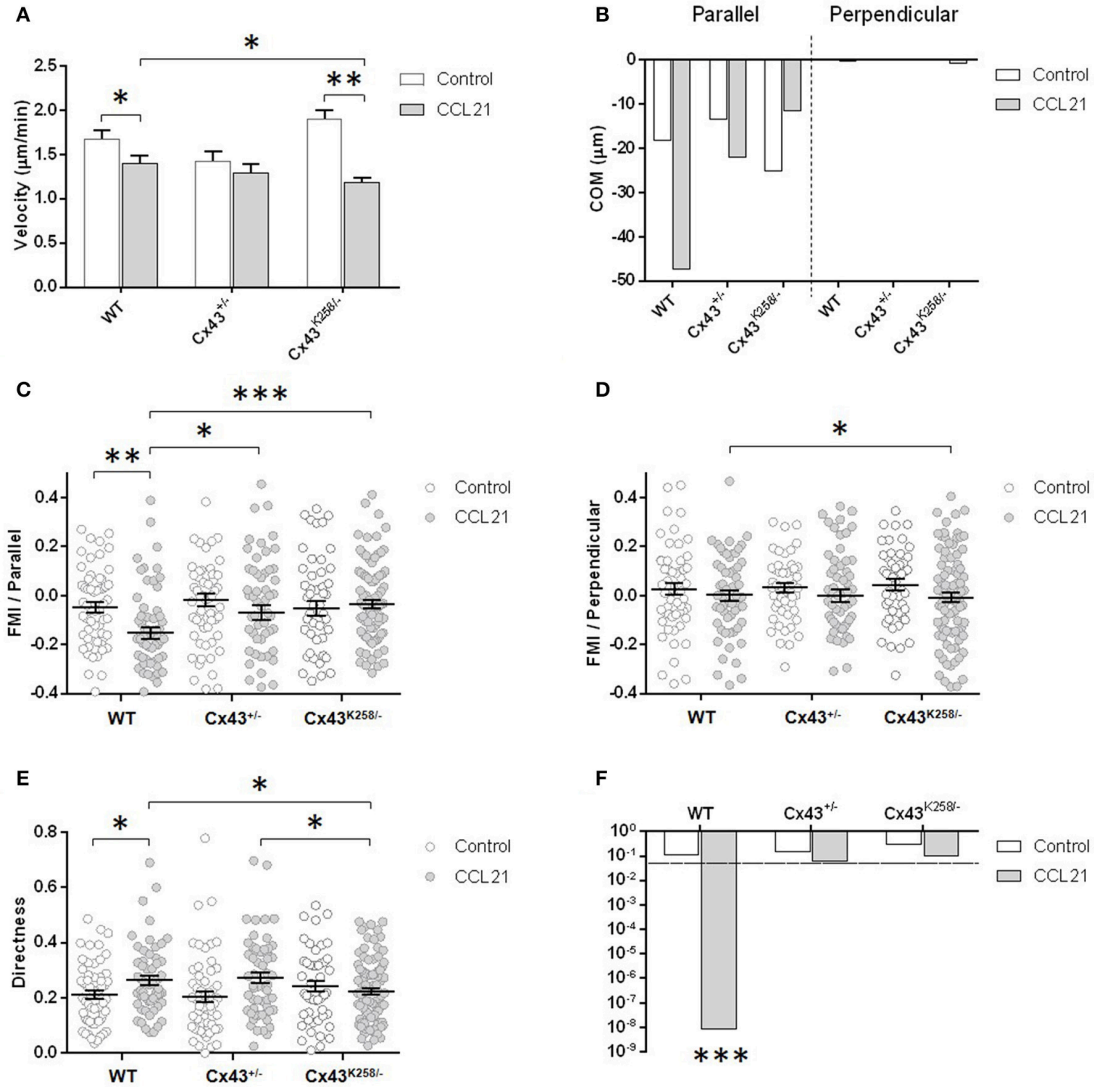
Quantitative analysis of the migratory behavior illustrated in Figures 4A–C, including velocity **(A)**, parallel and perpendicular COM **(B)**, parallel **(C)** and perpendicular FMI **(D)**, directness **(E)** and *p*-value of the Rayleigh test **(F)**, of WT, Cx43^+/−^ and Cx43^K258/−^ LPS-activated BMDCs. BMDCs from Cx43 mutant mice display altered directed migration in response to CCL21. CCL21: 200 ng/ml. Duration of the experiment: 3.5 h. Impaired directed migration of Cx43^K258/−^ BMDCs was observed in 3 independent experiments. ^*^p < 0.05 was considered significant; ^**^p < 0.01; ^***^p < 0.001.

We next evaluated the migratory behavior of BMDCs from mice with altered Cx43 expression. WT, Cx43^+/−^ and Cx43^K258/−^ BMDCs were subjected to an *in vitro* migration assay after activation with LPS for 24 h. In this experiment, BMDCs were seeded in μ-Slide chemotaxis^3D^ chambers and exposed to a gradient of CCL21 (200 ng/ml, decreasing from left to right) while monitoring their migration by time-lapse imaging for 3.5 h. Whereas, the majority of WT BMDCs migrated toward the CCL21 gradient within the course of the experiment (toward the left along the X axis as compared to the Y axis, Figure 4A), this movement was reduced in Cx43^+/−^ BMDCs (Figure 4B) and fully inhibited in Cx43^K258/−^ BMDCs (Figure 4C). Analysis of the migratory behavior was performed for parameters testing speed and directionality (velocity, parallel and perpendicular FMI, directness, *p*-value of the Rayleigh test) on these populations of migrating BMDCs as well as for the resulting displacement of their center of mass (COM), parallel and perpendicular to the CCL21 gradient (Figure 5). Velocity of BMDCs with different Cx43 genotypes was weakly affected although the one of CCL21-stimulated Cx43^K258/−^ BMDCs was lower than the one of WT BMDCs (Figure 5A). Despite the decrease in velocity, CCL21 strongly attracted WT BMDCs, as indicated by COM displacement (Figure 5B and “+” symbol on migration plots in Figure 4A) and by the increase in the negative parallel FMI value (Figure 5C) accompanied by an almost null perpendicular FMI (Figure 5D) relative to the gradient. In addition, directness (Figure 5E) and Rayleigh test (Figure 5F) indicate, respectively, that migration of WT BMDCs followed rather straightforward trajectories, and that these cells were significantly guided toward the CCL21 gradient. In contrast, Cx43^+/−^ and Cx43^K258/−^ BMDCs showed decreased COM, FMI, directness (Cx43^K258/−^ BMDCs only), and Rayleigh test, indicating reduced guidance toward the CCL21 gradient (Figures 5B–F). Reduced guidance of Cx43^K258/−^ BMDCs toward a 100 ng/ml CCL21 gradient was also observed after 75 min of migration while longer exposure to CCL21 was needed to detect an altered migratory behavior of Cx43^+/−^ BMDCs. These results indicate that BMDCs from Cx43 mutant mice display intrinsic altered migration in response to CCL21 mainly due to a defect in following the direction given by the CCL21 gradient.

**Figure 6:**
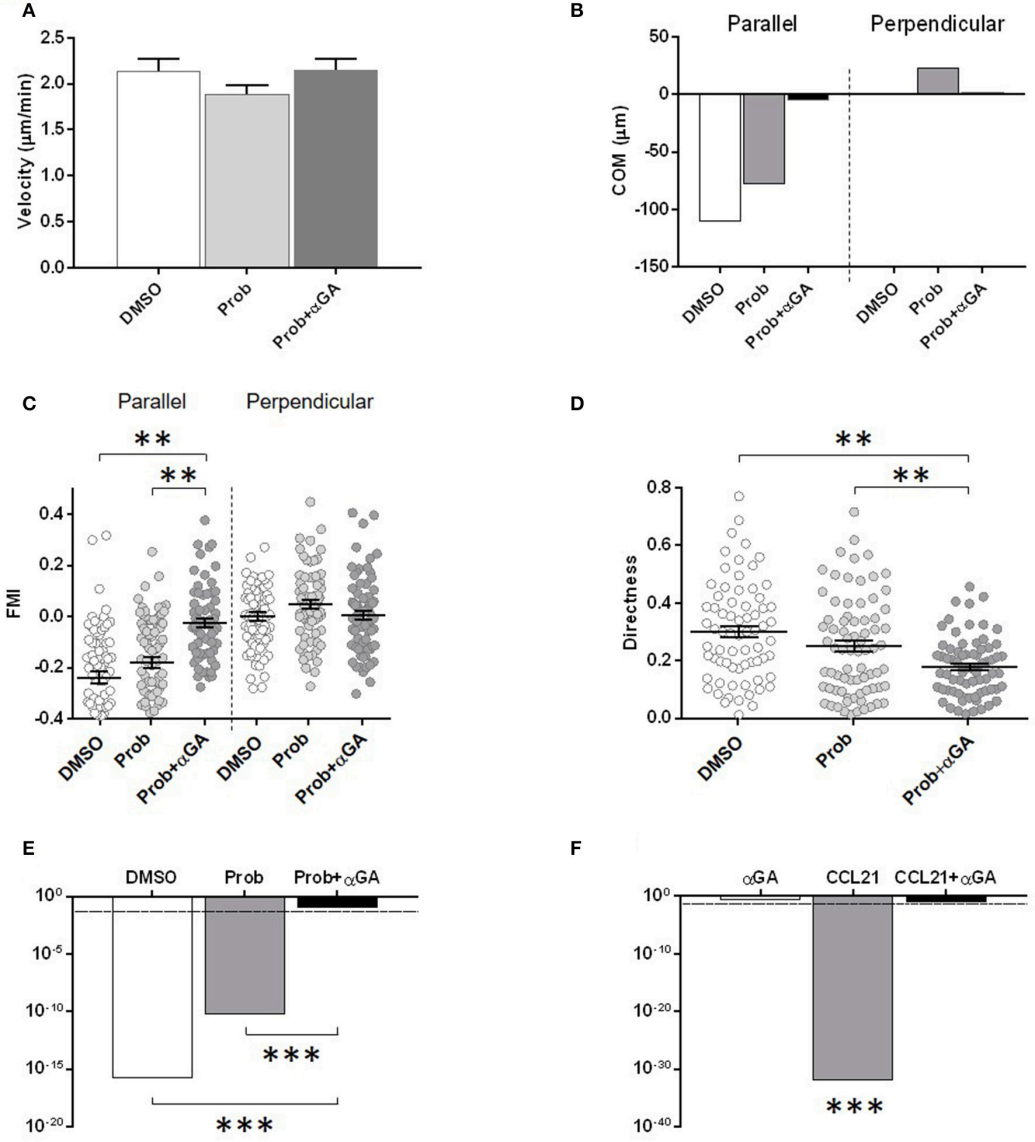
Quantitative analysis of the migratory behavior illustrated in Figures 4D–F, including velocity **(A)**, parallel and perpendicular COM **(B)**, parallel and perpendicular FMI **(C)**, directness **(D)** and *p*-value of the Rayleigh test **(E)**, of WT LPS-activated BMDCs exposed to DMSO, Probenecid (Prob) or Prob +α GA. DMSO was used as vehicle. Prob+αGA but not Prob alone altered the directed migration of WT BMDCs in response to CCL21. Probenecid: 2 mM; αGA: 100 μM. **(F)** *p*-value of the Rayleigh test for the migration of WT BMDCs subjected or not to CCL21 and in the presence or not of αGA. CCL21: 200 ng/ml. αGA: 100 μM. Duration of the experiment: 3.5 h. Data are representative of 2 independent experiments. ^*^p < 0.05 was considered significant; ^**^p < 0.01; ^***^p < 0.001.

Cx43 forms connexons at the surface of DCs that may circumstantially open and release nucleotides like ATP to the extracellular medium; this mechanism can also be fulfilled by pannexons made of Pannexin1 (Panx1) (Sosinsky et al., 2011). We first verified pannexin expression by qPCR in BMDCs from the Cx43 mutant mice. Panx1 mRNA was found in WT, Cx43^+/−^ and Cx43^K258/−^ BMDCs to similar extent whereas Panx2 and Panx3 were not detectable (data not shown). Next, we evaluated whether Panx1 channels contribute to the migratory behavior of BMDCs toward the CCL21 gradient and performed additional time-lapse recording of WT BMDCs in the presence of the Panx1 inhibitor probenecid. Probenecid did not interfere with the migration of BMDCs (Figures 4D,E, 6A–E). Importantly, addition of the connexin channel inhibitor 18 alpha-glycyrrhetinic acid (αGA) to the probenecid condition (Prob + αGA) markedly reduced the directed migration of WT BMDCs toward the CCL21 gradient without affecting their velocity (Figures 4F, 6A–E). Similar results were observed with αGA alone (Figure 6F). These results implicate that the activity of Cx43–made connexons, but not that of Panx1-made pannexons contributes to BMDC migration.

## Discussion

CCR7-mediated immune cell migration is critical for guiding DCs into lymph nodes. Immune cells enter lymph nodes either from the blood circulation or from peripheral tissues, such as the skin, *via* afferent lymphatics (Girard et al., 2012). Here, we studied the function of Cx43, a gap junction protein expressed in BMDCs. Our data indicate that Cx43 was not necessary for BMDC maturation, activation or induction of T-cell proliferation. However, it was needed for DC migration both *in vivo*, from peripheral sites to secondary lymphoid tissues, and *in vitro* using BMDCs subjected to a gradient of the CCR7-ligand CCL21. This function correlated with the structural expression of the Cx43 C-terminus, which appeared to be needed for CCR7-dependent migration.

Little is known about the mechanisms by which Cx43 influences the immune response. Cx43 has been detected in DCs and found to support homo- and heterocellular coupling between DCs and between DCs and B lymphocytes in secondary lymphoid organs (Krenács and Rosendaal, 1995; Rajnai et al., 2015). Evidence for a role of gap junctional intercellular communication in facilitating transport of Ag epitopes between cells, which in turn can be presented on MHCI and be recognized by cytotoxic T lymphocytes, has been reviewed (Glass et al., 2015). Recently, intestinal macrophages were found to collect soluble Ags from the gut lumen and transfer them on MHCII in DCs present in the lamina propria in a Cx43-dependent manner (Mazzini et al., 2014). Using a model of skin-derived DC migration after epicutaneous hapten sensitization, we added a piece of information by showing that Cx43 regulates the migration of primed DCs into draining lymph nodes. The emigration of DCs from the skin toward draining lymph nodes of Cx43^+/−^ and Cx43^K258/−^ mice was drastically reduced as compared to WT DCs.

Early *in vitro* data showed that inhibition of functional expression of Cx43 with pharmacological inhibitors, mimetic peptides or siRNA suppressed DC maturation and activation in response to cytokines, chemokines and PAMPs (Matsue et al., 2006; Corvalàn et al., 2007; Elqueta et al., 2009). We did not observe Cx43 dependence for maturation and activation of BMDCs derived from Cx43^+/−^ and Cx43^K258/−^ mice, as indicated by comparable levels of CD11c, CD86, CD80, CD40, MHC-II, and CCR7 surface markers. These results are in agreement with the report by Mazzini and collaborators who found normal Ag presenting cell function of mouse DCs with conditional deletion of Cx43 *in vitro* (Mazzini et al., 2014). We also observed *ex-vivo* Ag-dependent T-cell activation by BMDCs from our two genetically modified Cx43 mouse models. Both mouse strains, whereby Cx43^+/−^ mice are known to express half of the amount of Cx43 and Cx43^K258/−^ mice to express Cx43 channels that lack post-translational regulation, have revealed phenotypic alterations in cartilage structure (Gago-Fuentes et al., 2016) and inflammation (Sarieddine et al., 2009; Kozoriz et al., 2010; Morel et al., 2016). Thus, genetic manipulations of Cx43 do not confirm observations made in DCs treated with connexin blockers and indicate that Cx43 expression is not prerequisite for their Ag presenting cell function (Glass et al., 2015).

Whereas BMDCs with altered functional expression of Cx43 retained their ability to mature and activate T-cells *ex-vivo*, they failed, however, to migrate toward a CCL21 gradient in an *in vitro* assay. CCL21 slightly decreased the velocity of WT BMDCs but directed their migration along the chemotactic gradient. In contrast, the migration of Cx43^+/−^ and especially of Cx43^K258/−^ BMDCs was reduced due to strong decrease in directed migration. This phenotype was not caused by impaired expression or ability of mutant BMDCs to respond to CCR7 activation, at least in terms of Ca^2+^ mobilization. Importantly, inhibition of migration was observed in WT BMDCs exposed to αGA, a connexin channel inhibitor, but not to probenecid, a Panx1 channel blocker. This observation is consistent with the report of Molica and collaborators where the migration of DCs to draining lymph nodes after epicutaneous hapten sensitization was comparable in Panx1^−/−^ and control mice (Molica et al., 2017). These results suggest that Cx43-made connexons likely contributes to the control of mDC motility and opens up toward specific translational possibilities.

Recently, the motility of immune cells was shown to rely on CCR7-mediated signals via c-Src tyrosine kinase in response to CCL21 (Hauser et al., 2016). The C-terminus domain of Cx43 interacts with key protein partners, including c-Src (Giepmans et al., 2001; Toyofuku et al., 2001; Sorgen et al., 2004), which are involved in cytoskeleton rearrangement, cell motility (Behrens et al., 2010; Machtaler et al., 2014; Chen et al., 2015; Ambrosi et al., 2016; González-Sánchez et al., 2016) and Cx43 channel activity (Giepmans et al., 2001; Lin et al., 2001). Thus, alteration in Cx43 structure and expression may not only disrupt cell movement but also uncouple CCR7 activation from Cx43-made connexon opening. For instance, interaction of the C-terminus with the cytoplasmic domain of Cx43 makes connexons available to open in response to a trigger (Wang et al., 2013a,b). Thus, the number of connexons that open in Cx43^+/−^ BMDCs exposed to CCL21 would be reduced whereas connexons lacking their C-terminal tails would not be able to open at all. This, in turn, would decrease ATP leakage and paracrine/autocrine activation of purinergic receptors. Downstream activation of hemichannels to modulate cell adhesion and/or migration via purinergic signaling has been previously reported in leukocytes (Wong et al., 2006; Chen et al., 2010; Lohman et al., 2015; Sáez et al., 2017) and astrocytes (Alvarez et al., 2016). Although the links between Cx43-made connexon activity and directional motility of BMDCs remain to be established, our results point to Cx43 as a potential novel partner in DC chemotaxis.

## Author contributions

RR, JD, AZ, and MB performed the research. FM did the experiment shown in Figure 1B. SH, FM, RR, and BK participated to the design of some experiments and corrected the paper. MC designed the research and wrote the paper.

### Conflict of interest statement

The authors declare that the research was conducted in the absence of any commercial or financial relationships that could be construed as a potential conflict of interest.
