# Supplementary figures and images for: Dendritic Cell Migration Toward CCL21 Gradient Requires Functional Cx43
Source: Front Physiol. 2018 Mar 27;9:288. doi: 10.3389/fphys.2018.00288 (PMC5880903; doi:10.3389/fphys.2018.00288)

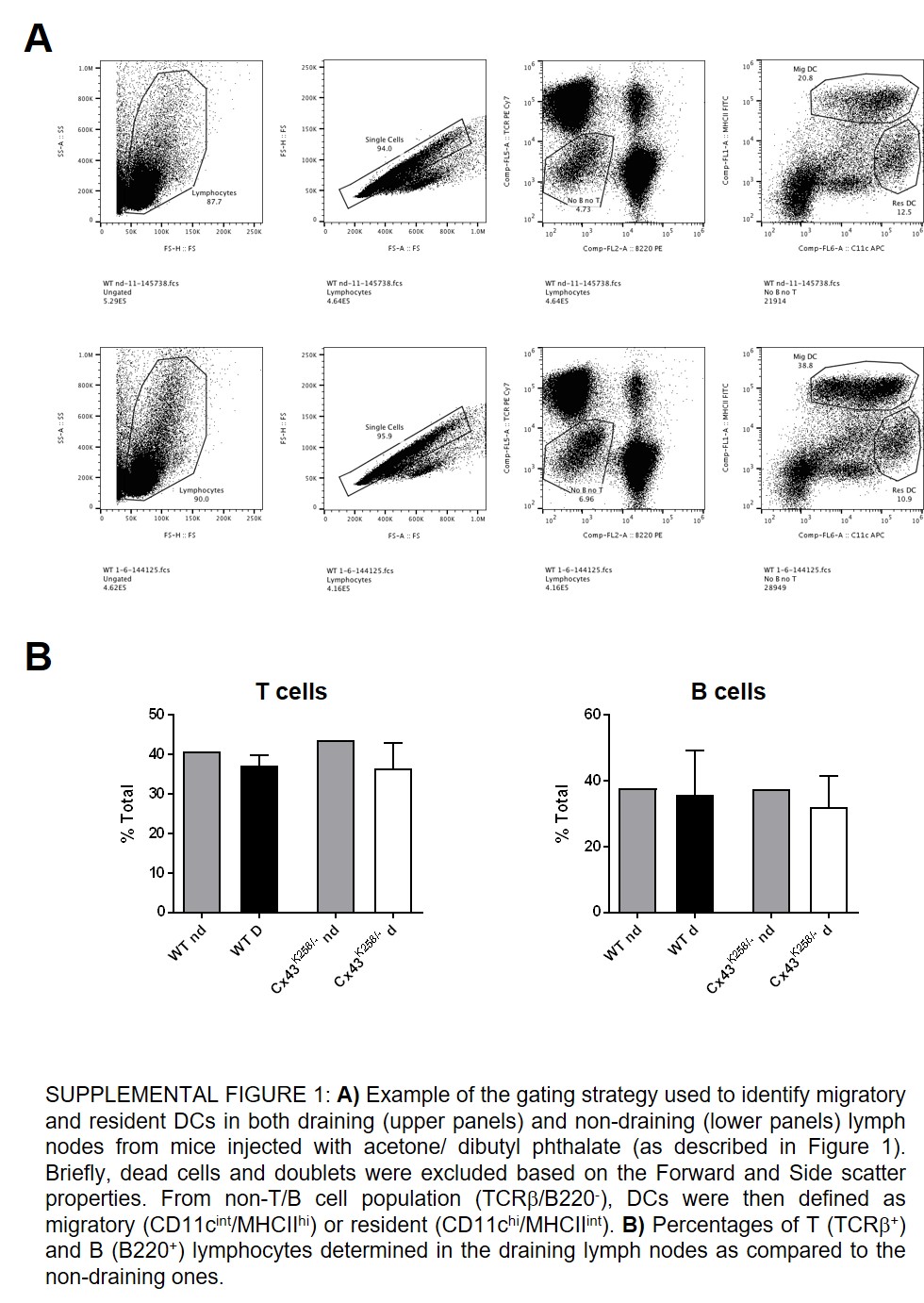

Supplement: Supplementary file 1 [file Image1.JPEG]

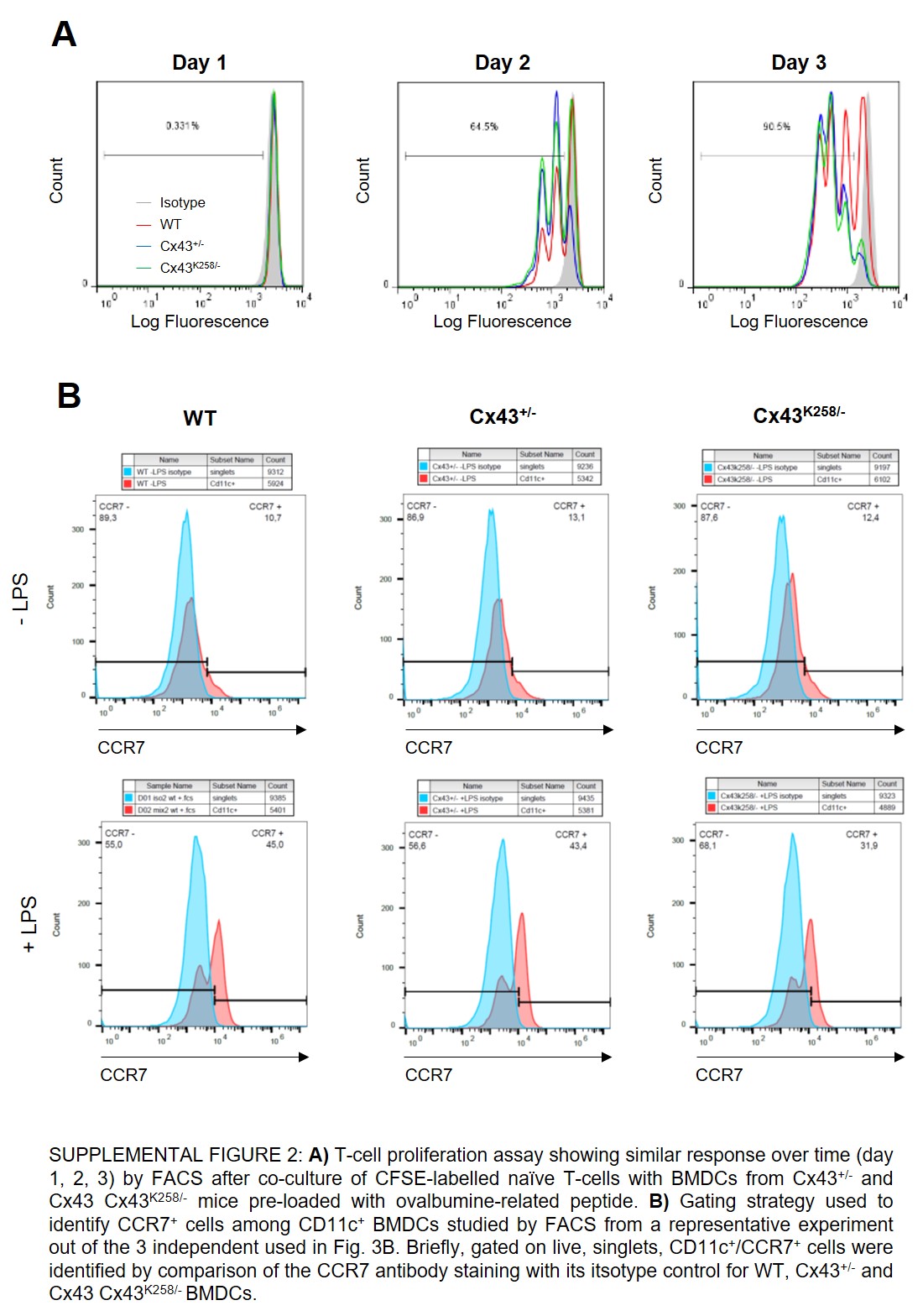

Supplement: Supplementary file 2 [file Image2.JPEG]
